# Effects of a Formula-Based Ketogenic Diet on Refractory Epilepsy in 1 to 3 Year-Old Patients under Classic Ketogenic Diet

**Published:** 2019

**Authors:** Parvaneh KARIMZADEH, Toktam MOOSAVIAN, Hamid Reza MOOSAVIAN

**Affiliations:** 1 Pediatric Neurology Research Center, Research Institute for Children’s Health, Shahid Beheshti University of Medical Sciences, Tehran, Iran.; 2 Pediatric Neurology Department, Mofid Children’s Hospital, Faculty of Medicine, Shahid Beheshti University of Medical Sciences, Tehran, Iran; Department of Clinical Pathology, Faculty of Veterinary Medicine, University of Tehran, Tehran, Iran; 3 Department of Paediatric Neurology, Faculty of Medicine, Child Growth and Development Research Center, Isfahan University of Medical Sciences, Isfahan, Iran.; 4 Department of Clinical Pathology, Faculty of Veterinary Medicine, Tehran University of Medical Sciences, Tehran, Iran

**Keywords:** Ketogenic diet, Refractory seizure, Children, Powder ketogenic formula

## Abstract

**Objectives:**

The classic ketogenic diet (CKD) as a potential epilepsy treatment with high-fat has not good tolerability in some patients, and so many families refuse to use this diet for long term especially in children younger than 2 year. In the present study, the efficacy and tolerability of the CKD only diet were compared with CKD combined a formula-based powder in children between 1 and 3 yr with intractable epilepsy.

**Materials & Methods:**

We *randomly* enrolled 45 children referred to Mofid Children’s Hospital, Tehran**, **Iran from April 2016 to May 2017 with refractory epilepsy for CKD only (control group), and formula based CKD (experimental group) treatment. Subjects were followed up for at least six months.

**Results:**

Most of the patients in CKD only group did not tolerate the diet and were reluctant to eat homemade foods with high fat. All families of the patients younger than 2 yr old in this group chose to discontinue CKD and pursued other options. About 33% of the families of the patients younger than 2 yr old and 41.6% of the total patients between 1-3 yr old in experimental group stayed to the end of the trial, and all of them showed more than 90% reduction in seizure frequency after 6 months. Moreover, regardless of the other variables, using formula increased the chance of responding to treatment 7.32 times.

**Conclusion:**

A ketogenic diet using a powder ketogenic formula is effective, safe, and tolerable in infants and children with refractory seizure especially for younger patients who are reluctant to eat ketogenic homemade foods.

## Introduction

Epilepsy is the most common neurologic disorder in children and 20%-25% of these patients are resistant to medical treatments which impose high expenses on the family and affects their quality of life (1,2). Other treatment options such as surgery need to have a special epileptic focus. In addition, most of the refractory seizures have genetic origins without any brain epileptic focus, and the surgery option is available only in a small number of countries (1-4). 

Ketogenic diet has been a well-known treatment for refractory seizures since 1972 with positive effects on patients’ social and cognitive abilities (3, 5-7). However, most of the studies about ketogenic diet have been designed for children over three years old or in a wide age range group without age categorization (8-12). Ketogenic formula powder is a commercial product that tastes similar to infantile formula. In the first days of starting ketogenic diet during which a child often refuses solid food, this formula is simply acceptable and is a suitable alternative (9). 

However, there are few studies about the efficacy of the respective diet in children especially children younger than 2 yr old. Therefore, we aimed to study and compare the tolerability and efficacy of classic ketogenic diet with the powder formula in children between 1 and 3 yr of age with intractable epilepsy. 

## Materials &Methods

Overall, 45 children, 12-36 months with refractory seizure, referred to Pediatric Neurology Department of Mofid Children’s Hospital (Major tertiary university-affiliated Child Neurology Center in Tehran, Iran) from April 2016 to May 2017 were enrolled in our study. 

The parents were informed about all objectives and details of the research. The exclusion and inclusion criteria were evaluated completely for each patient, and the cases were evaluated by trained nutritionist and skilled staff. 

This research was evaluated and approved by the Ethical Board Committee of Shahid Beheshti University of Medical Science, Tehran, Iran (IR.SBMU.REC.1396.1)

The inclusion criteria consisted of children between 11-36 months, with at least 2 episodes of refractory seizure per week, treated with three first-line antiepileptic medications for at least 3 months before admission. They should also have compliant parents. The exclusion criteria comprised metabolic diseases such as fatty acid oxidation disorders and primary carnitine deficiency, dilated cardiomyopathy, systemic disorders diabetes mellitus, and organic acidemia disorders. At first, a complete history was taken and physical examination was done. 

Urine organic acids measurement, serum and urine amino acids chromatography, acylcarnitine profile, ammonia, lactate, pyruvate and mass spectrophotometric-based metabolic acid measurements were performed to rule out any metabolic diseases. Brain MRI was done for each patient, and EEG was performed before admission. Twenty-one cases entered the classic ketogenic diet group without KetoCal® (CKD only) and 24 cases randomly entered the ketogenic KetoCal® formula group. As the primary step, after obtaining informed consent, children received 67 cc/kg water regimen. Electrolyte analysis, lipid profile, urine Ca/Cr ratio, and EEG were also ordered. Urine ketone was assessed in every urination episode, and blood sugar was measured three times a day. After urine ketone reached the desired amount (+2 to +3), the ketogenic diet was started with the following order: First day, one third of 70% of calculated calorie for age and gender. Second day, two third of 70% of calculated calorie for age and gender. Third day, total calorie in three meals. In the KetoCal® group, the patients ranging from1-2 yr old and 2-3 yr old received KetoCal® formula providing 50% and 30% of calculated calorie, respectively. 

The patients were followed up for 6 months. Complete medical evaluation was done by an expert neurologist and nutritionist in the 1^st^, 3^rd^, and 6^th ^months after admission. The parents were asked to record seizures frequency and report any complications. During the follow-up and interviews, if the seizure reduction was less than 50% of the baseline after three months, and in case of irreversible lethargy, pancreatitis, triglyceride level more than 1000 mg/dl, and persistent urolithiasis, the diet would be stopped and the patient would be excluded from the study.

Statistical analysis was performed using SPSS Statistical Software (ver. 22, Chicago, IL, USA), and Stata Statistical Software (ver. 13). Student’s *t*-test, chi-square, Fisher exact test, Mann-Whitney, and *McNemar test were used to compare the results*. *P*-value less than 0.05 was considered as statistically significant. 

## Results

All 45 cases admitted in the present study had neurological disabilities including different types of global developmental delays. 

The frequency of seizure, EEG changes, anti-epileptic medications, and the neurodevelopmental condition were evaluated in both groups. The demographic factors, age, gender, weight, and head circumference between two groups had no significant differences (*P*>0.05). Both groups were similar in terms of MRI results, EEG, triglyceride and cholesterol (or LDL) level, and random urine Ca/Cr ratio. The age difference between two groups were not statistically significant. 

The mean±standard deviation of the follow-up duration was 0.67±1.8 months in CKD only group and 3.08±2.69 months in KetoCal® group. The follow-up duration difference between the two groups was significant (*P*<0.05). 

The frequency of weekly seizures was assessed in 1^st^, 3^rd^, and 6^th ^month after intervention in both groups (Table 1). About 50% seizure reduction was more prominent in KetoCal® group than CKD only group, and the difference between the two groups was statistically significant (*P*<0.05). Seizure reduction between two groups was also compared based on the seizure type. In the KetoCal® group, the therapeutic response in the 3^rd^ and 6^th^ months of follow-up was quite remarkable in myoclonic seizures and infantile spasm, with a significant statistical difference (*P*<0.01) (Table 2). The effect of age, gender, and type of seizure on the therapeutic response were studied. Based on the results (Table 3), the 50%-reduction of seizure was significantly higher in individuals who were in KetoCal® group than in CKD only group (Odds ratio: 7.32, Confidence Interval: 2.27-23.58, *P*<0.05). In addition, other variables such as seizure type and age showed a significant effect on seizure reduction. Success rate was higher in the children with myoclonic seizure type when compared with other types of seizure (Odds ratio: 1.49, Confidence Interval: 1.09-2.08, *P*<0.05). Furthermore, the more the age of the patient, the more the therapeutic response (for each month increase in age, the therapeutic response was increased about 6%, with confidence limit of 1.005-1.11)

In the KetoCal® group at the time of admission, 1 out of 24 cases had normal EEG, while after 6 months of study, 6 out of 10 patients had normal EEG, and the difference was statistically significant (*P*<0.05). 

Since several cases were excluded from the CKD only diet group in the first month, we were not able to evaluate EEG in this group. 

At the time of admission, 21 out of 24 patients in the KetoCal® group had a moderate to severe cognitive developmental disorder based on Denver scoring, while after 6 months this ratio became 0.3, which was statistically significant (*P*<0.05).

 The excluded cases from the CKD only group were 90.5 %( 19 out of 21 cases) and in the KetoCal® group was 58.3% (14 out of 24 cases). The difference was statistically significant (*P*<0.05). After 6 months, 100% (11 out of 11 patients) and 61.7% (8 out of 13 patients) of the patients under 2 yr old were excluded from CKD only and KetoCal® groups, respectively, and the difference was statistically significant (*P*<0.05).

The complications observed in KetoCal® group included resistant urolithiasis in one patient and irreversible mental deterioration especially decreased cognition in two patients. All excluded cases from CKD only group between 1-2 yr old happened to be for the diet intolerability in the first week of admission. In KetoCal® group, 6 patients between the age of 2 to 3 yr were excluded from the study as one patient in the first week, 3 patients after one month, and 2 patients after three months. Seizure recurrence and/or diet intolerability were the main reasons for exclusion from the KetoCal® group. In CKD only group, 8 of 19 patients were excluded because of the lack of ketone production. However this was not seen in KetoCal® group. None of the patients were excluded from the study because of the lack of ketone production. None of the patients had biochemical disorders causing the ketogenic diet to be discontinued. The random urine Ca/Cr ration was more than 0.2 in 13 patients, treated with polycitrate-potassium.

**Table 1:** The number of patients who stayed in different groups in different months

Ketogenic diet	1 month	3 month	6 month
Classic diet without ketocal®	4(19%)	2(9.5%)	2(9.5%)
Classic diet with ketocal®	18(75%)	13(54.2%)	10(41.7%)
*P*-value	<0.001	0.002	0.02

**Table 2:** The number of patients that their seizures decreased to less than half after diet, based on the seizure type

Ketogenic Diet	Seizure Type	Time		
		1 Month	3 Month	6 Month
Classic diet without Ketocal®	Infantile spasm and myoclonus	2(16.7%)	1(8.3%)	1(8.3%)
	Others	2(22.2)	1(11.1)	1(11.1)
*P*-value		0.59	0.69	0.69
Classic Diet with Ketocal®	Infantile spasm and myoclonus	12(58.7)	10(71.4)	9(64.3)
	Others	6(60)	3(30)	1(10)
*P*-value		0.19	0.05	0.01

**Table 3:** The effect of age and sex in treatment response

Variable	OR	15%CLOR	*P*-value
Ketocal® diet	7.32	(2.27-23.58)	0.001
Seizure type :myoclonic	1.49	(1.09-2.08)	0.013
Sex	0.78	(0.27-2.28)	0.65
Age	1.06	(1.005-1.11)	0.04

## Discussion

CKD as a potential epilepsy treatment is a high-fat, low carbohydrate diet and is strongly considered in children who failed two or three antiepileptic drugs, regardless of the age (13-15). In addition, it is the diet of choice for the *treatment *of diseases such as pyruvate dehydrogenase deficiency and glucose transporter protein 1 (GLUT-1) deficiency syndrome (15-18). However, CKD as a high-fat diet has not good tolerability in some patients, and many families refuse to use the diet for the long term especially in children younger than 2 yr old. Many prospective studies that studied the effect of CKD in the treatment of epilepsy in long term have been performed in patients older than 2 yr old (19).

In the present study, the efficacy and tolerability of CKD only and formula-based CKD with KetoCal® 4 to 1 powder have been studied in children with intractable epilepsy. Most patients in CKD only group did not tolerate the diet and were reluctant to eat homemade food with high fat. All families of the patients younger than 2 yr old in this group chose to discontinue CKD and pursued other options. On the other hand, 33% of the families with patients younger than 2 yr old in KetoCal® group remained to the end of the trial. Although in comparison with CKD only group, more patients remained in KetoCal® group, a significant percentage of families in KetoCal® group also discontinued the diet. At the beginning of the study, the children fasted in the hospital for 72 h, and it was probably one of the major causes of family dissatisfaction. Therefore, the authors suggested removing the fasting period or shortening it. In this case, 3:1 diet can be used to gradually increase blood ketone levels and ‏obtain appropriate ketonuria. Our results indicate that ketogenic diet using the powder formula such as KetoCal® was palatable and tolerable and can be a suitable diet especially for infants and children who are reluctant to eat homemade food. These results are in line with the results of a previous study that showed the efficacy and tolerability of KetoCal® in children with refractory seizures (8). Overall, the diet of 59% of parents was palatable and tolerable enough. In the present study, the response rate to the ketogenic diet was age-related as with increasing every month of age the response rate to treatment increased by 6%. However, ketogenic diet was more effective in younger patients. The researchers believe that production and utilization of ketone bodies in younger patients are better than older patients (20, 21). On the other hand, the response rate to the ketogenic diet was not age-related (10). We found that in the younger patients, not only the tolerability of the ketogenic diet was poor, but also the efficacy of the diet was low. Compared to older patients, the increase of ketone bodies was not desirable in younger patients on ketogenic diet, and in case of boosting ketone bodies, the seizures were not well controlled. In older patients, the efficacy and tolerability of the ketogenic diet were better. 

The duration of follow-up had a statistically significant difference (*P*<0.05) between CKD only group (0.67 months) and KetoCal® group (3.08 month), and most patients in CKD only group did not tolerate the diet and discontinued the trial. The percentage of patients who had a greater than 50%-reduction of seizures after 1-3 and 6 months was higher in KetoCal® group in comparison with CKD only group (*P*<0.05). Regardless of the other variables, the KetoCal® diet increased the chance of responding to treatment 7.32 times. The efficacy and suitable tolerability of the ketogenic diet were showed in 38 infants and children between 3 months and 5 yr old with epileptic encephalopathies using ketogenic diet as KetoCal® formula alone or 80% of the daily caloric amount (10). All the children initiating the diet continued the diet for 1 month, and 92%, 73.7%, and 52.7% of them continued the diet for at 3 months, 6 months and 1 year, respectively. They indicated that 28.9 % of children had a greater-than-50%-reduction of seizures and 23.7% were seizure-free during a 12-months follow-up period. In a research study, one-third of the patients on the ketogenic diet showed more than 90% reduction in seizure. However, completely seizure-free subjects were rare (22). In another study, KetoCal® was used for children between 12 months and 5 yr old with refractory epilepsy who were reluctant to eat homemade food (8). During a 4-month follow-up the median seizure frequency per week was reduced in 68.2% of patients, 40.9% showed a 50%-90% reduction in seizure frequency per week, and 27.3% showed more than 90% reduction in seizure frequency per week. Myoclonic seizure was a good prognostic factor as the patients with myoclonic seizure reacted favorably to the diet in comparison to other patients. The response rate to diet in the patients with infantile spasms, myoclonic, atonic, and tonic-clonic generalized seizures was better in comparison to complex partial seizures (23). 

**In conclusion,** a ketogenic diet using a powder formula (KetoCal®) is effective, safe, and tolerable in infants and children with refractory seizures. *Response rate *to ketogenic diet is age-related. The KetoCal® diet can be an appropriate choice especially for those younger than 2 yr old and patients who are reluctant to eat homemade food. 
